# Preoperative intra-aortic balloon counterpulsation in cardiac surgery: propensity analysis of data obtained from the ARIAM Registry of Cardiac Surgery

**DOI:** 10.1186/cc14277

**Published:** 2015-03-16

**Authors:** MD Delgado-Amaya, C Joya-Montosa, J Muñoz-Bono, E Curiel-Balsera

**Affiliations:** 1 Hospital Regional de Málaga, Spain

## Introduction

The aim of our study was to assess whether the preoperative use of IABP is beneficial in patients undergoing cardiac surgery of any kind.

## Methods

An observational, retrospective, multicenter study of all patients undergoing cardiac surgery and included in the ARIAMANDALUCIA Registry of Cardiac Surgery from March 2008 to July 2012. The probability of placing IABP in the preoperative period has been calculated, making a propensity analysis to obtain two homogeneous groups treated with or without the IABP, based on personal history, functional status and type of surgery. Seventy-seven patients with preoperative IABP were matched with 77 patients without BCIAO with the nearest propensity score. We used the chi-square test or Student *t *test as needed and binary logistic regression for multivariate analysis so we can rule out possible confounding variables. We used the statistical package R v2.12 for MAC.

## Results

A total of 8,026 were recorded, in 77 of them an IABP was inserted before the surgery.We performed a propensity score analysis by pairing 72 patients with and without BCIAO based on epidemiological factors and type of surgery. In the analysis of all-cause 30-day mortality, 27% of patients in whom IABP was inserted prior surgery died versus 13.1% of patients without IABP preoperative implantation (*P *= 0.043) A combined endpoint that included need for prolonged mechanical ventilation over 24 hours or reoperation or mediastinitis or stroke after surgery or 30-day mortality was performed and occurred in 58.3% of patients with preoperative IABP versus 41.7% without it (*P *= 0.046). When stratified by preoperative risk (analyzed with EuroSCORE), no difference between groups was observed (*P *= 0.62, OR 0.75 (0.23 to 2.35)) for mortality rate and (*P *= 0.11, OR 0.47 (0.19 to 1.18)) for the combined endpoint. The patients with preoperative IABP implantation had a higher ICU length of stay (10.6 ± 7.7 vs. 4.6 ± 6.7, *P *= 0.046) with no differences in terms of overall hospital stay (21.8 ± 18.7 vs. 18.9 ± 22.08, NS).

## Conclusion

The use of IABP prior to cardiac surgery in patients at high risk does not reduce the mortality rate nor the combined endpoint described above. ICU length of stay was greater in those patients in whom IABP was implanted prior to surgery; there were no differences in overall hospital stay.

